# pH Homeostasis and Sodium Ion Pumping by Multiple Resistance and pH Antiporters in *Pyrococcus furiosus*

**DOI:** 10.3389/fmicb.2021.712104

**Published:** 2021-08-16

**Authors:** Dominik K. Haja, Michael W. W. Adams

**Affiliations:** Department of Biochemistry and Molecular Biology, University of Georgia, Athens, GA, United States

**Keywords:** *Pyrococcus furiosus*, hyperthermophile, Mrp antiporter, pH homeostasis, sodium ions

## Abstract

Multiple Resistance and pH (Mrp) antiporters are seven-subunit complexes that couple transport of ions across the membrane in response to a proton motive force (PMF) and have various physiological roles, including sodium ion sensing and pH homeostasis. The hyperthermophilic archaeon *Pyrococcus furiosus* contains three copies of Mrp encoding genes in its genome. Two are found as integral components of two respiratory complexes, membrane bound hydrogenase (MBH) and the membrane bound sulfane sulfur reductase (MBS) that couple redox activity to sodium translocation, while the third copy is a stand-alone Mrp. Sequence alignments show that this Mrp does not contain an energy-input (PMF) module but contains all other predicted functional Mrp domains. The *P. furiosus* Mrp deletion strain exhibits no significant changes in optimal pH or sodium ion concentration for growth but is more sensitive to medium acidification during growth. Cell suspension hydrogen gas production assays using the deletion strain show that this Mrp uses sodium as the coupling ion. Mrp likely maintains cytoplasmic pH by exchanging protons inside the cell for extracellular sodium ions. Deletion of the MBH sodium-translocating module demonstrates that hydrogen gas production is uncoupled from ion pumping and provides insights into the evolution of this Mrp-containing respiratory complex.

## Introduction

Multiple resistance and pH adaptation (Mrp) antiporters are multisubunit complexes that couple transport of Na^+^ (or K^+^) ions across the membrane to the proton motive force (PMF; Ito et al., 2017). They are part of a larger group of Na^+^/H^+^ antiporters including eukaryotic NHE families (Brett et al., 2005) and bacterial NhaA families (Lentes et al., 2014) and are the only subgroup that is encoded by a multi-gene operon. Mrp complexes perform a number of different physiological roles, including maintaining pH and Na^+^ homeostasis, Na^+^ tolerance, pathogenesis, and arsenic tolerance (Kudo et al., 1990; Hamamoto et al., 1994; Padan and Schuldiner, 1996; Kosono et al., 2005; Padan et al., 2005; Kashyap et al., 2006). The typical *mrp* operon is composed of a seven gene *mrpABCDEFG* cluster, where each gene is predicted to encode a membrane protein. Mrp complexes can be further divided into three groups depending on the arrangement of the A and B genes (Swartz et al., 2005; Ito et al., 2017). Group 1 *mrp* operons encode separate *mrpA* and *mrpB* genes, with both *mrpB* and *mrpA* containing an *mrpB* domain. Group 2 *mrp* operons lack the *mrpB* gene, but *mrpA* contains two *mrpB* domains, representing a fusion of *mrpB* and *mrpA*. Group 3 *mrp* operons are characterized not by the presence or absence of *mrpB* domains but by the lack of an *mrpA* gene, containing only *mrpD*, sometimes with multiple copies; Group 3 Mrp complexes also include those found as part of larger respiratory complexes. The consequences of the differences in operon structure between the three subgroups of Mrp complexes have not been well studied. The proteins encoded by *mrpA* and *mrpD* are homologous to each other and are both predicted to contain H^+^ pumps (Morino et al., 2017). Homologs of MrpA/D are found in a number of different respiratory complexes, ranging from bacterial and mitochondrial complex I to the membrane bound hydrogenase (MBH) and membrane bound sulfane sulfur reductase (MBS) of the hyperthermophilic archaeon *Pyrococcus furiosus*. These complexes are proposed to have evolved from the fusion of an Mrp-like membrane antiporter with a cytoplasmic NiFe hydrogenase and couple ion pumping to cytoplasmic redox activity. Structures of complex I, MBH, and MBS have all been recently determined (Sharma et al., 2009; Baradaran et al., 2013; Yu et al., 2018, 2020).

Until recently, Mrp complexes were studied primarily through mutagenesis and homology modeling using respiratory complexes as templates. However, two Mrp structures, the group 1 Mrp from *Anoxibacillus flavithermus* and the group 2 Mrp from *Dietzia* sp. DQ12-45-1b, were very recently determined (Li et al., 2020; Steiner and Sazanov, 2020). While the subunit organization between the two complexes differs as described above, the overall structures are very similar. The major difference between the two complexes lies in a highly negatively charged cavity between MrpA and MrpF in the *A. flavithermus* Mrp, proposed to be part of the Na^+^ translocation path, that is absent in the *Dietzia* Mrp, suggesting that the Na^+^ pathways are distinct between various Mrp complexes.

*Pyrococcus furiosus* is a hyperthermophilic archaeon isolated from a marine environment with an optimal growth temperature of 100°C (Fiala and Stetter, 1986). Its genome encodes three copies of a group 3 Mrp complex. Two copies are found in the operons encoding MBH and MBS, which, in the absence and presence of elemental sulfur, couple reduction of protons and polysulfide, respectively, to Na^+^ pumping across the membrane (Sapra et al., 2000; Wu et al., 2018). The resulting Na^+^ gradient is used by a Na^+^-dependent ATP synthase to produce ATP (Pisa et al., 2007). The third operon encodes a stand-alone Mrp and contains *mrpEFGBB’CD*, where *mrpB* and *mrpB*’ are homologous to group 1 and 2 *mrpB*. Interestingly, this operon lacks the *mrpA* gene, which is proposed to encode a proton-translocating pump and to be the primary driving force for Na^+^ pumping by the remaining subunits. For example, the MrpA subunit from *Bacillus subtilis* has been found to be essential for Na^+^-dependent pH homeostasis, with various mutations showing no PMF-dependent Na^+^ efflux (Ito et al., 1999). In the MBH complex, MrpA has been replaced by the membrane-anchored hydrogenase domain, and Na^+^ pumping is driven by coupling redox activity, rather than by PMF as in Mrp (Yu et al., 2018). The operon encoding the stand alone Mrp is unique in that it does not contain any energy-input module. However, the operon retains genes encoding a single proton-pumping subunit, as well subunits that contain the predicted Na^+^ channel, suggesting that this Mrp complex retains its role in either pH or Na^+^ sensing or homeostasis. While various models for coupling the PMF to ion pumping in Mrp have been proposed, the exact mechanism remains unknown (Li et al., 2020; Steiner and Sazanov, 2020). The lack of an energy-input module represents an opportunity to compare *P. furiosus* Mrp to those that contain the additional MrpA subunit and could provide valuable insight into the coupling mechanism of the PMF to ion pumping.

There are multiple examples of organisms that contain multiple Mrp gene clusters. For example, *Staphylococcus aureus* has two sets of group 1 *mrp* gene clusters, one encoding a Na^+^/H^+^ antiporter and the other encoding an Mrp of unknown function (Swartz et al., 2007). The marine bacterium *Oceanobacillus iheyensis* has two sets of *mrp* gene clusters, while *Microbacterium* sp. TS-1 has three *mrp* gene clusters, two belonging to group 2 and the third to group 3. However, their physiological functions remain unknown (Fujinami et al., 2013; Krulwich and Ito, 2013). The marine hyperthermophile *Thermococcus onnurineus* NA1, a close relative of *P. furiosus*, contains three group 3 *mrp* gene clusters, although all three are associated with respiratory complexes (Lim et al., 2010).

In this study, we have used deletion strains to determine the physiological functions of two of the Mrp complexes in *P. furiosus*, the stand-alone Mrp and the Mrp that is part of MBH. While many organisms contain multiple copies of the *mrp* gene cluster, *P. furiosus* is unique in that it contains operons encoding both a stand-alone Mrp as well as ones associated with two respiratory complexes. This allows for a direct comparison of the roles of Mrp in various types of complexes, as well as providing insights into the different functions of the three Mrp subgroups.

## Materials and Methods

### Preparation of Liquid and Solid Medium

The naturally competent *P. furiosus* strain COM1 was used to delete *Pf1147-53* (*mrpEFGBB’CD*). *P. furiosus* transformants were grown in defined maltose media as previously described (Lipscomb et al., 2011). The medium for *P. furiosus* growth was composed of 1x base salts, 1x trace minerals, 1x vitamin solution, 2x 19-amino-acid solution, 0.5% (wt/vol) maltose, 10 μM sodium tungstate, and 0.25 mg/ml resazurin, with added cysteine at 0.5 g/L, sodium sulfide at 0.5 g/L, sodium bicarbonate at 1 g/L, and 1 mM sodium phosphate buffer (pH 6.8). The 5x base salts stock solution contained (per liter) 140 g of NaCl, 17.5 g of MgSO_4_·7H_2_O, 13.5 g of MgCl_2_·6H_2_O, 1.65 g of KCl, 1.25 g of NH_4_Cl, and 0.70 g of CaCl_2_·2H_2_O. The 1,000x trace mineral stock solution contained (per liter) 1 ml of HCl (concentrated), 0.5 g of Na_4_EDTA, 2.0 g of FeCl_3_, 0.05 g of H_3_BO_3_, 0.05 g of ZnCl_2_, 0.03 g of CuCl_2_·2H_2_O, 0.05 g of MnCl_2_·4H_2_O 0.05 g of (NH_4_)_2_MoO_4_, 0.05 g of AlK(SO_4_)·2H_2_O, 0.05 g of CoCl_2_·6H_2_O, and 0.05 g of NiCl_2_·6H_2_O. The 200x vitamin stock solution contained (per liter) 10 mg each of niacin, pantothenate, lipoic acid, *p*-aminobenzoic acid, thiamine (B_1_), riboflavin (B_2_), pyridoxine (B_6_), and cobalamin (B_12_) and 4 mg each of biotin and folic acid. The 25x 19-amino-acid solution contained (per liter) 3.125 g each of arginine and proline; 1.25 g each of aspartic acid, glutamine, and valine; 5.0 g each of glutamic acid and glycine; 2.5 g each of asparagine, histidine, isoleucine, leucine, lysine, and threonine; 1.875 g each of alanine, methionine, phenylalanine, serine, and tryptophan; and 0.3 g tyrosine. A solid medium was prepared by mixing an equal volume of liquid medium at a 2x concentration with 1% (wt/vol) Phytagel (Sigma) previously autoclaved to solubilize, and both solutions were maintained at 95°C just prior to mixing. The medium was poured into glass petri dishes immediately after mixing.

### Assembly of the Knock-In Cassette

The knock-in cassette used for the deletion of Mrp contains 500 bp flanking regions, amplified from *P. furiosus* genomic DNA, for the upstream and downstream flanking region (UFR and DFR, respectively), and the selection marker (*pyrF*-P_gdh_, a uracil biosynthetic gene that allows for nutritional selection of transformants), amplified by using pGL021 as the template (Lipscomb et al., 2011). The knock-in cassette was assembled using overlapping PCR (Supplementary Figure S1; Bryksin and Matsumura, 2010) and was checked for the correct length on a 1% agarose gel. Supplementary Table S1 contains primers used in this study.

### Deletion of *mrp*

Aliquots of *P. furiosus* culture typically grown to mid-log phase (2 × 10^8^ cells/ml) in defined liquid medium containing 20 μM uracil were mixed with knock-in cassette DNA at a concentration of 2–10 ng DNA per μl of culture, spread in 30 μl aliquots onto defined solid medium containing 20 μM uracil. Plates were placed inverted in anaerobic jars and incubated at 90°C for ~64 h. Colonies were picked into 4 ml of defined medium lacking uracil in Hungate tubes and incubated anaerobically overnight at 90°C. The genomic DNA isolated by Zymobead Genomic DNA Kit (Zymo Research) was used for PCR screening, which was carried out by using GXL polymerase (Takara, ClonTech). PCR screening was performed using a pair of primers outside the *mrp* locus in order to confirm that the transformation cassette recombined into the correct locus based on the length of the PCR product. After PCR confirmation of a deletion, the resulting strain was passaged twice on solid medium lacking uracil for colony purification. Upon colony purification, the PCR screening product was sent for confirmation using Sanger sequencing (Genewiz, South Plainfield, NJ), and the resulting strain MW0576 saved as a glycerol stock and subsequently used as the parent for the double deletion of *mrp* and *Pf1423-25* (*mbhABC*).

### Double Deletion of *mrp* and *mbhABC*

5-fluoroorotic acid (FOA) counterselection was used to remove the selection marker from MW0576. 5-FOA is converted to a toxic product by the protein encoded by the *pyrF* gene, so only cultures that spontaneously lose the *pyrF* gene are able to grow, enabling a second round of nutritional selection using uracil (Lipscomb et al., 2011). Around 30 μl of culture was plated directly onto solid medium plates containing 8 mM 5-FOA and 20 μM uracil. Plates were placed inverted in anaerobic jars and incubated at 90°C for ~64 h. Colonies were picked into 4 ml of defined medium in Hungate tubes containing 20 μM uracil and incubated anaerobically overnight at 90°C. The resulting strain was passaged twice on solid medium for colony purification, and the transformation was repeated as described above using *Pf1422* and *Pf1426* as the UFR and DFR of the knock-in cassette, respectively (Supplementary Figure S1). All strains used and created in this study are listed in Table 1.

**Table 1:** Strains generated and used in this study.

Trivial name	Lab name	Phenotype	Source
Parent	MW0003	Δ*pyrF*::*pyrF*	Lipscomb et al., 2011
ΔMrp	MW0576	Δ*mrpEFGBB’CD*::*P_gdh_pyrF*	This work
ΔMbhABC	MW0574	Δ*mbhABC*::*P_gdh_pyrF*	Yu et al., 2018
ΔMrp/ΔMbhABC	MW0582	Δ*mbhABC*Δ*mrpEFGBB’CD*::*P_gdh_pyrF*	This work

### Growth Studies

Strains were grown in defined maltose media in 50 ml cultures contained in 100 ml bottles at 90°C with continuous shaking. Where indicated 50 mM 3-(N-morpholino) propanesulfonic acid (MOPS; pH 6–8) was added to the growth medium. About 1 ml culture samples were taken at desired time intervals. pH was measured using an Orion Dual Star pH/ISE meter (Thermo Scientific). Cell protein was measured using the Bradford method. H_2_ production was measured by taking 1 ml headspace samples from growing cultures and analyzed in a 6850 Network Gas Chromatograph (Agilent Technologies).

### Preparation of Cell Suspensions and H_2_ Production Assays

Strains were grown in defined maltose media in 1 L culture bottles at 90°C with shaking. Cells were harvested by centrifugation at 18,000 × *g* for 10 min in a Beckman-Coulter Avanti J-30i centrifuge. Cell suspensions were created by washing harvested cells with an anaerobic resuspension buffer containing 20 mM imidazole, 30 mM MgCl_2_·6H_2_O, 0.5 M KCl, 2 mM cysteine-HCl, pH 6.5 and resuspending them in the same buffer at cell densities of OD_600_ = 0.6. H_2_ production assays are modified from that reported previously (Lim et al., 2014). Cell suspensions (final volume, 2 ml) were added to rubber-sealed glass 8 ml vials and the headspace was flushed with argon. Samples were incubated at 80°C for 3 min and the reaction was initiated by the addition of the desired concentration of NaCl from an anaerobic 2 M stock solution. At various time intervals, gas samples were taken and analyzed in a 6850 Network Gas Chromatograph (Agilent Technologies).

### Multiple Sequence Alignments

Amino acid sequences were aligned using the Clustal Omega alignment tool in the Uniprot database using default parameters (Consortium TU, 2020). Similarity of amino acids was based on the properties of the side chains as determined by Uniprot. UniprotKB ID numbers used for the alignments are shown in Supplementary Table S2.

## Results and Discussion

### *Pyrococcus furiosus* Mrp Is Missing an Energy-Input Module but Contains Conserved Ion Transport Pathways

**Figure 1:**
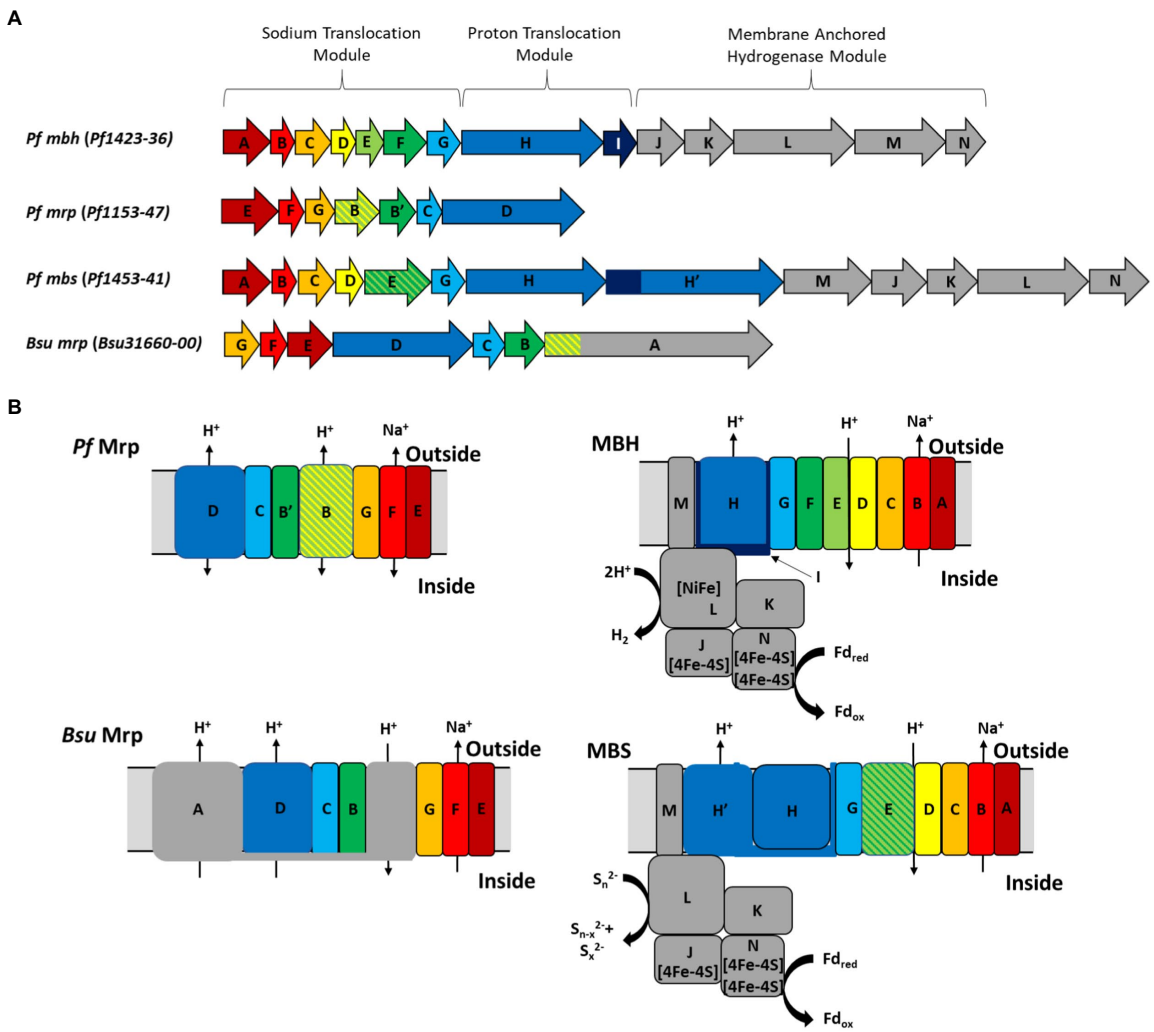
**(A)** Operon structure and **(B)** models of *P. furiosus* Mrp, MBH, and MBS and *B. subtilis* Mrp. Colors represent gene homology based on *Pf* MBH. The different MBH modules are labeled as described in the text. Black arrows represent predicted ion translocation pathways.

Amino acid sequence alignments (Supplementary Figure S2) derived from the seven *P. furiosus mrp*-related genes (Figure 1A) with the homologous genes in the *mbh*, *mbs*, and *B. subtilis mrp* operons provide insights into the structure and function of the stand-alone Mrp complex. A full comparison of all *P. furiosus* Mrp, MBH and MBS subunits, as well as the *B. subtilis* Mrp, is shown in Table 2. First, *P. furiosus* MrpB and MrpB’ are homologous to transmembrane (TM) helices 17–21 of *B. subtilis* MrpA (containing one copy of the MrpB domain) and *B. subtilis* MrpB, respectively. The two MrpB domains are homologous to the MbhD and E subunits, which form the secondary proton pathway at the junction between the proton pumping module and the sodium translocation module. Additionally, *P. furiosus* MrpB is homologous to a fusion of MbhD and MbhE, while MrpB’ is homologous to MbhF. The reason for this fusion is unknown, but *P. furiosus* MbsE (of MBS) represents a fusion of MbhE and MbhF (of MBH). These subunit fusions are additional evidence that the subunit composition of the various Mrp groups is not as important to the function of the complex as the presence of the required domains. Importantly, *P. furiosus* Mrp does not contain an MrpA homolog, nor does it contain any energy-input modules, and the driving force behind ion pumping by the complex therefore remains unknown. Finally, several of the amino acids proposed to be involved in both H^+^ pumping and Na^+^ ion translocation pathways are highly conserved (based on the properties of the relevant amino acid side chains) between *P. furiosus* Mrp, MBH, and MBS suggesting that Mrp plays a role in H^+^/Na^+^ translocation. Models of *P. furiosus* Mrp, MBH, and MBS and *B. subtilis* Mrp based on the alignments are shown in Figure 1B.

**Table 2:** Subunit homology of *Pyrococcus furiosus* membrane bound hydrogenase (MBH), membrane bound sulfane sulfur reductase (MBS), and multiple resistance and pH (Mrp) and *Bacillus subtilis* Mrp.

Proposed MBH module	*Pyrococcus furiosus* MBH complex	*Pyrococcus furiosus* MBS complex	*Bacillus subtilis* Mrp complex	*Pyrococcus furiosus* Mrp Complex
Membrane-anchored hydrogenase module	MbhJ	MbsJ	-	-
	MbhK	MbsK	-	-
	MbhL	MbsL	-	-
	MbhN	MbsN	-	-
	MbhM	MbsM	-	-
-	-	MbsH’ N-terminal TM1-14	MrpA TM1-16	-
-	MbhI N-terminal^a^	MbsH’ C-terminal TM 15-16	-	-
-	MbhI C-terminal^b^		-	-
Proton translocation module	MbhD^c^	MbsD	MrpA TM17-21	MrpB
	MbhE^c^	MbsE N-terminal		
	MbhG	MbsG	MrpC	MrpC
	MbhH	MbsH	MrpD	MrpD
Sodium translocation module	MbhF	MbsE C-terminal	MrpB	MrpB’
	MbhA	MbsA	MrpE	MrpE
	MbhB	MbsB	MrpF	MrpF
	MbhC	MbsC	MrpG	MrpG

### Deletion of the Sodium-Pumping Module of MBH Leads to a Decreased Growth Rate

**Figure 2:**
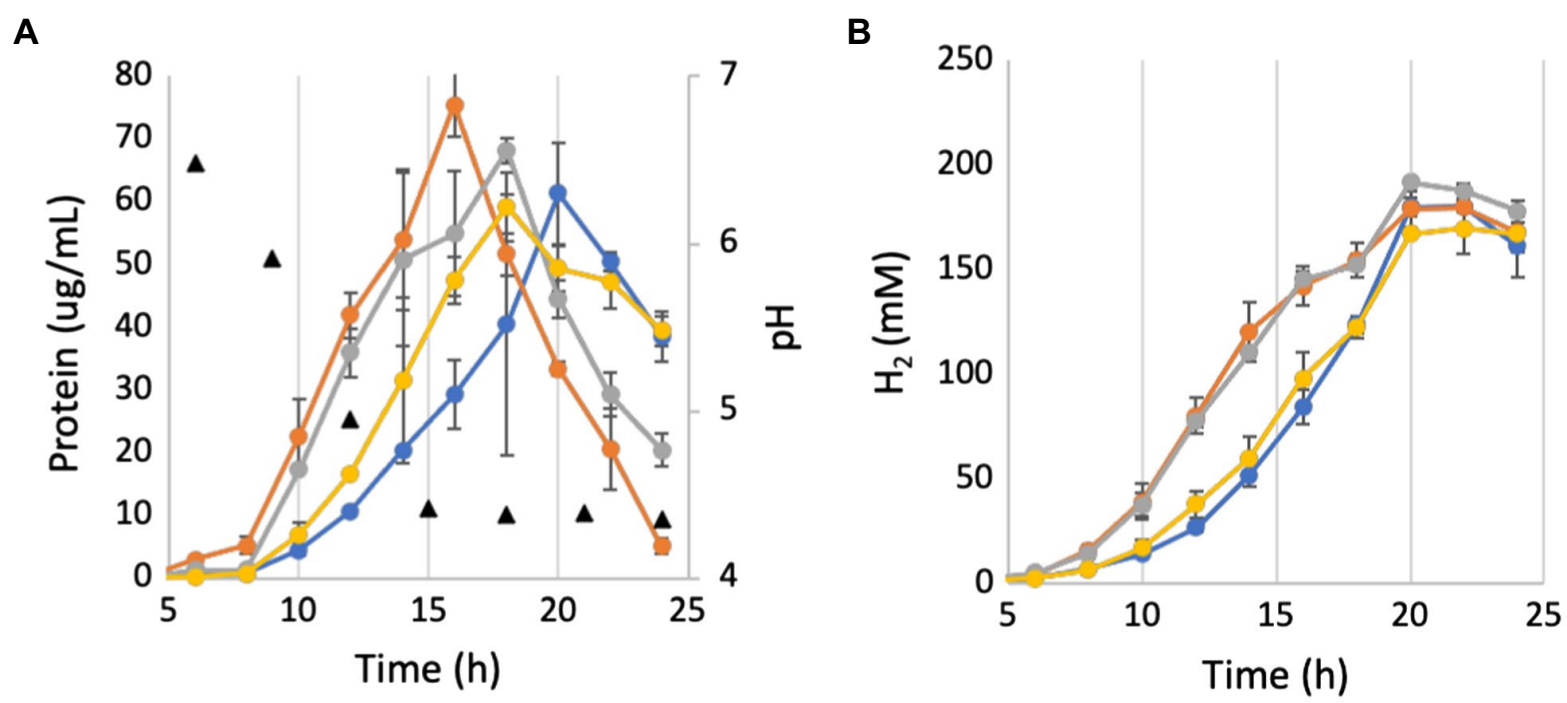
Growth and pH **(A)** and H_2_ production **(B)** of deletion strains in unbuffered medium. Yellow, parent strain; blue, ΔMbhABC; orange, ΔMrp; and gray, ΔMrp/ΔMbhABC. Black triangles represent average measured pH values. Error bars represent SD of biological triplicate samples.

**Figure 3:**
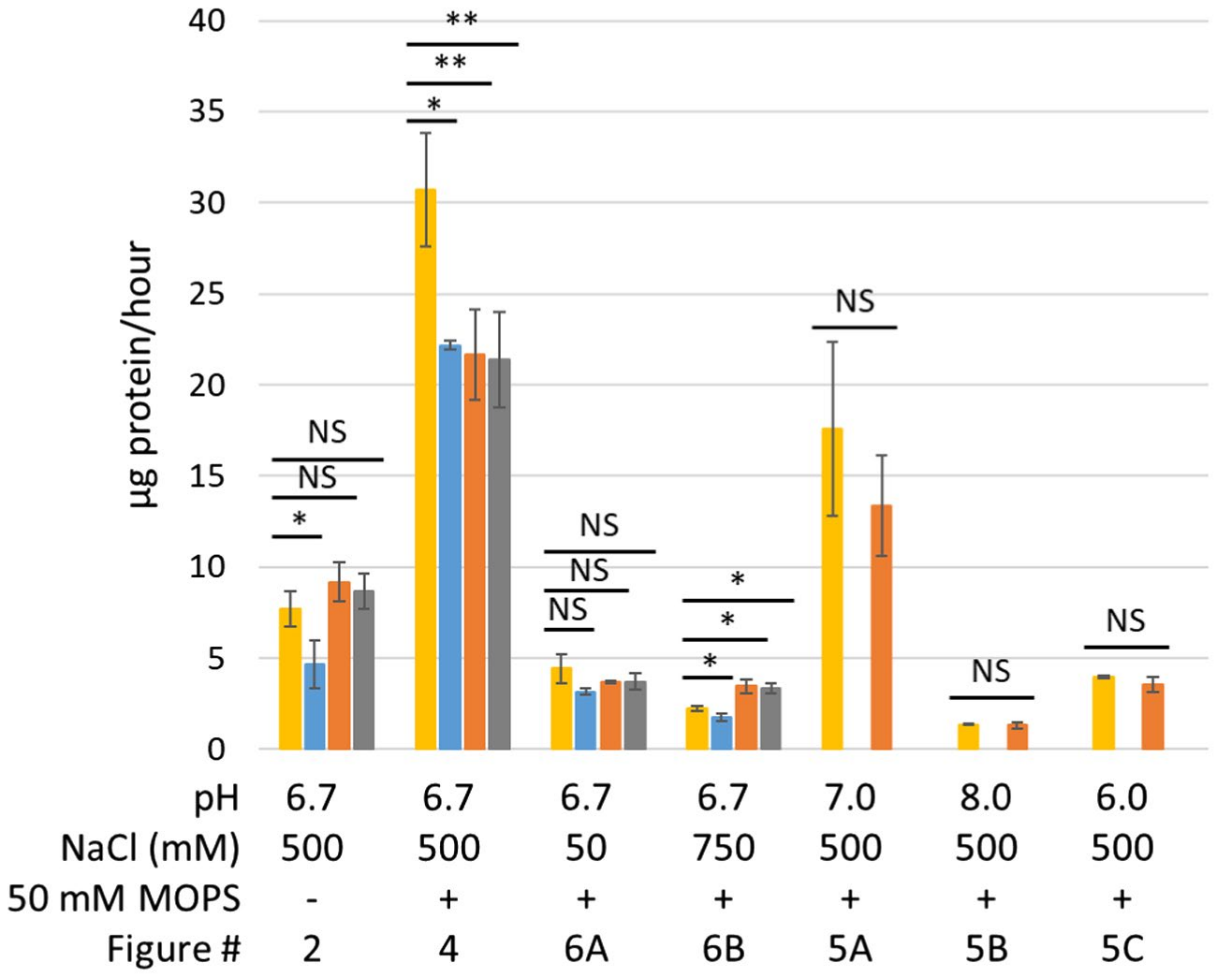
Average growth rates from growth data presented in this manuscript. Yellow, parent strain; blue, ΔMbhABC; orange, ΔMrp; and gray, ΔMrp/ΔMbhABC. Error bars represent SD of biological triplicate samples. A two-tailed *t*-test assuming unequal variance was used to determine statistical significance. NS, not significant; ^*^p < 0.05; ^**^p < 0.01.

The operon structures encoding *P. furiosus* MBH and Mrp are shown in Figure 1A. MBH can be divided into three modules: the membrane-anchored hydrogenase module, which includes the NiFe active site in MbhL, the proton translocation module, and the sodium translocation module. MBH catalyzes the oxidation of reduced ferredoxin, a low molecular weight iron-sulfur protein that is the primary electron carrier in the cell, and the reduction of protons to generate H_2_ (Sapra et al., 2000). The redox activity in the hydrogenase module is coupled to conformational changes in the proton translocation module that generates a H^+^ gradient. This H^+^ gradient is exchanged for a Na^+^ gradient by the sodium translocation module (Figure 1B; Yu et al., 2018). A Δ*mbhABC* deletion strain, referred to as ΔMbhABC, was shown to have diminished Na^+^-dependent H_2_ production activity using cell suspension assays, demonstrating that the Na^+^ ion pathway is located in MbhABC (Yu et al., 2018; Supplementary Figure S5). The cell suspension assays are independent of glycolysis and can be used to indirectly measure Na^+^ ion pumping activity; the ΔMbhABC strain retained ~60% of Na^+^-dependent activity, suggesting that there are other Na^+^-translocating complexes in the cell that contribute to the ion gradient. Additionally, the specific activity of hydrogenase-catalyzed H_2_ production in membrane fractions was unchanged between ΔMbhABC and the parent strain, suggesting that while redox activity is used to drive ion pumping, MBH retains this activity whether or not additional sodium is pumped across the membrane by the sodium translocation module (Yu et al., 2018). Without Na^+^ pumping driving additional ATP synthesis by the Na^+^-dependent ATP synthase in *P. furiosus*, ATP is only generated through glycolysis in ΔMbhABC. This difference in energy yield is reflected in the statistically significant decreased growth rate of ΔMbhABC compared to the parent (4.6 + 1.3 vs. 7.7 + 1.0 μg protein/h; *p* = 0.016; Figures 2A, 3). Maximum cell yield (based on intracellular protein concentrations, used for all growth experiments) and H_2_ production (Figure 2B) is unchanged between the two strains.

### *Pyrococcus furiosus* Mrp Is Involved in the pH Response of the Cell

The homology between the subunits of *P. furiosus* Mrp and MBH suggests that Mrp also contains Na^+^ translocating activity; hence, a Δ*mrp* strain, referred to as ΔMrp, was generated to determine the effects of Mrp on Na^+^ translocation in *P. furiosus*. The ΔMbhABC strain, as well as a double deletion strain (ΔMrp/ΔMbhABC) were used to compare the Na^+^ pumping effects between the two Mrp-containing complexes. All strains were first grown in a defined maltose medium with a “normal” NaCl concentration of 500 mM, typical for a marine microorganism like *P. furiosus* (Figure 2A). Both ΔMrp and the double deletion strain appear to have a faster, but statistically insignificant, growth rate than the parent strain (9.2 + 1.0 and 8.7 + 0.9 μg protein/h, respectively). Specific hydrogen production rates were similar for all strains (0.1 + 0.01 mM H_2_/g protein/h; Supplementary Figure S3). The downstream end products of glycolysis in *P. furiosus* are H_2_, acetate, and CO_2_, and production of the latter causes a rapid drop in the pH of the growth medium (from a starting pH of 6.7–4.4 within 15 h; Mukund and Adams, 1995). At low pH, cells begin to lyse, increasing soluble protein but decreasing total cellular protein; cell lysis is therefore represented by a drop in total cellular protein concentrations. As anticipated, both the ΔMrp and ΔMrp/ΔMbhABC strains appear to be more sensitive to changes in pH than the parent strain. The pH of the medium was the same between the four strains at each sampled timepoint (Figure 2A), showing that the faster growth rate of ΔMrp and ΔMrp/ΔMbhABC does not lead to faster acidification of the medium and suggesting that the increased growth rate is not a result of increased glycolytic activity.

### *Pyrococcus furiosus* Shows an Increased Growth Rate Under Ideal pH Conditions When Mrp Is Present

**Figure 4:**
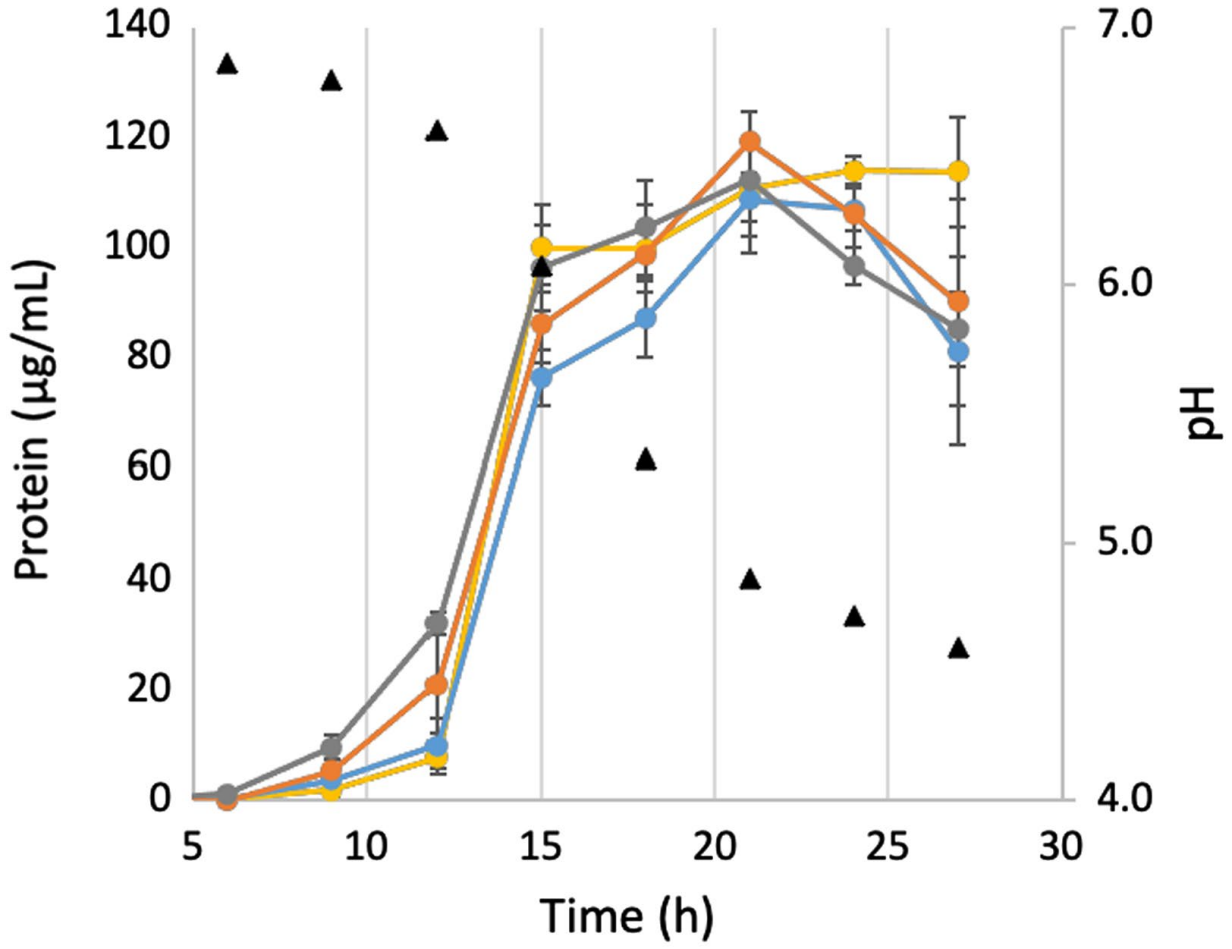
Growth and pH of deletion strains in buffered medium. Yellow, parent strain; blue, ΔMbhABC; orange, ΔMrp; and gray, ΔMrp/ΔMbhABC. Error bars represent SD of biological triplicate samples.

In order to investigate the effects of pH on growth, the growth in 500 mM NaCl was repeated in the presence of 50 mM MOPS buffer in order to lessen the effect of CO_2_ production on medium pH. As seen in Figure 4, while the buffer does keep the pH constant for longer, it still begins to drop once cells enter into late exponential phase. However, while cells in the buffered medium still begin to lyse after the pH drops, buffering the medium prevents the fast pH-induced lysis of the ΔMrp and ΔMrp/ΔMbhABC seen in Figure 2A. Additionally, while there is no difference in maximum cell density (~120 μg/ml) between any of the strains, the parent strain has a significantly faster growth rate (30.7 + 3.1 μg protein/h) than any of the other strains (21.4–22.2 μg protein/h; *p* = 0.008–0.02).

**Figure 5:**
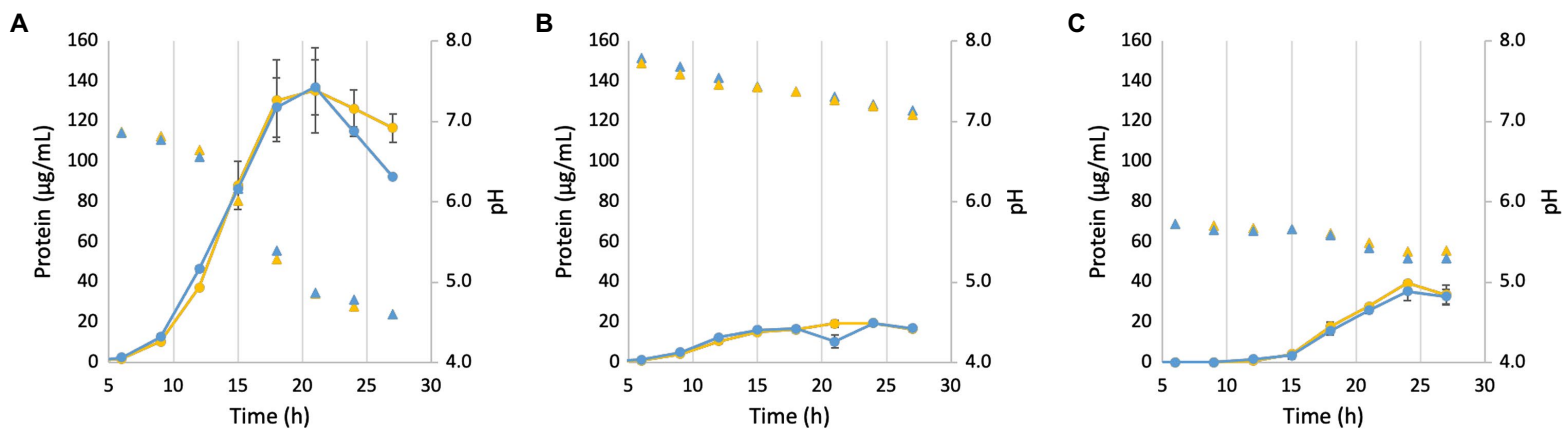
Growth and pH of the parent (yellow) and ΔMrp (blue) strain at different starting pH values. **(A)**: pH 7.0; **(B)**: pH 8.0; and **(C)**: pH 6.0. Filled circles represent protein concentration, while filled triangles of the same color represent the corresponding pH. Error bars represent SD of biological triplicate samples.

In order to test the role of Mrp in the pH response, the ΔMrp and parent strains were grown under varying initial pH regimes (Figure 5). When the pH was raised (to pH 8.0; Figure 5B) from the pH for optimum growth (pH 7.0; Figure 5A), both strains grew poorly, only reaching ~15% of the maximum protein yield at optimum pH. When the pH was lowered (pH 6.0; Figure 5C), both strains showed a longer lag phase (~15 h), and the maximum protein yield was only ~30% that of cells grown at the optimum. The change in pH (ΔpH = 1.0 and 0.5 for cells grown at pH 8 and 6, respectively) was insignificant under suboptimal pH conditions, and neither strain underwent cell lysis upon reaching stationary phase. However, while the pH stays relatively stable through 12 h when starting at the optimum pH of 7.0, it drops rapidly (ΔpH = 2.4) when cells reach stationary growth phase. Once the pH drops to ~4.5, the ΔMrp strain begins to lyse more rapidly than the parent. Both strains produce similar concentrations of acetate during growth (equivalent to CO_2_ production, which leads to the change in pH), suggesting that product formation is not different between the strains and that a different response to pH is the sole contributor to cell lysis (Supplementary Figure S4). These data together suggest that the *P. furiosus* Mrp does not increase the optimal pH range that supports growth but is involved in the pH response.

### *Pyrococcus furiosus* Mrp Uses Sodium as a Coupling Ion to Maintain Cytoplasmic pH

**Figure 6:**
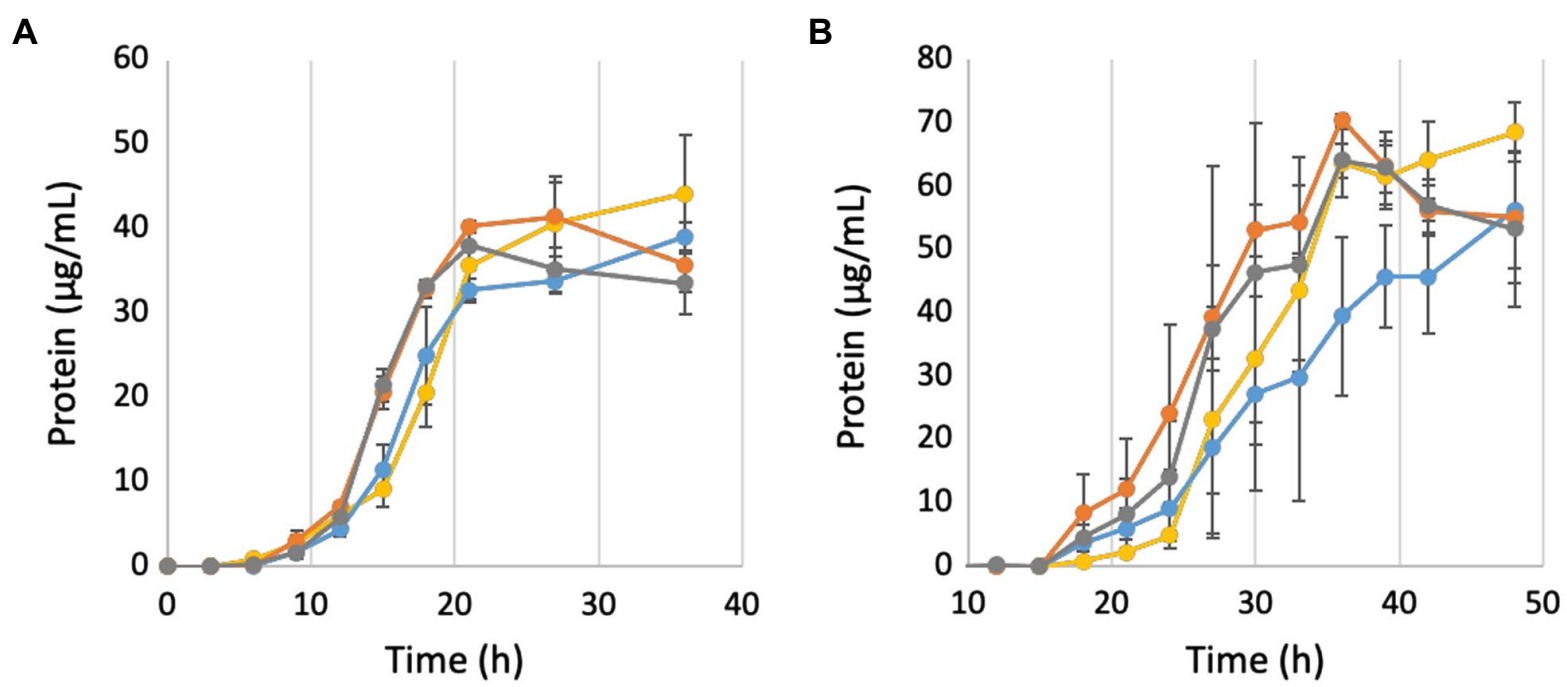
Growth of deletion strains in buffered medium containing 50 mM **(A)** and 750 mM **(B)** NaCl. Yellow, parent strain; blue, ΔMbhABC; orange, ΔMrp; and gray, ΔMrp/ΔMbhABC. Black triangles represent average measured pH values. Error bars represent SD of biological triplicate samples.

To further investigate the role of Mrp in Na^+^ ion translocation in response to changing pH, all strains were grown under both low (50 mM; Figure 6A) and high (750 mM; Figure 6B) NaCl concentrations. When the Na^+^ concentration is decreased from 500 to 50 mM, the parent strain no longer grows faster than the ΔMbhABC strain (4.4 + 0.8 and 3.2 + 0.2 μg protein/h, respectively), suggesting that at low sodium ion concentrations MBH is no longer able to generate a sufficient gradient for additional ATP synthesis. Additionally, the ΔMrp and ΔMrp/ΔMbhABC strains no longer grow faster than the parent strain (3.7 + 0.1 and 3.7 + 0.5 μg protein/h), suggesting that the faster growth rates seen under “normal” Na^+^ concentrations are due to changes in Na^+^ flux through ATP synthase when Mrp is deleted. At a higher Na^+^ concentration (750 mM), all strains showed a negative growth phenotype compared to “normal” 500 mM NaCl conditions (~50% of the maximum cell yield); no growth was observed in any strains at 1 M Na^+^ (data not shown). Taken together, the data show that the presence of Mrp in the cells does not significantly expand the range of Na^+^ concentrations suitable for growth but rather that sufficient Na^+^ is required in order for Mrp to affect growth of *P. furiosus*.

As the pH in the cell drops, Mrp likely maintains the cytoplasmic pH at the physiological value by pumping protons out of the cell at the cost of the Na^+^ gradient. Under ideal pH conditions, Mrp operates in the reverse direction, instead contributing to the Na^+^ gradient. In order to confirm that Na^+^ is the coupling ion used by Mrp, Na^+^-dependent H_2_ production assays were performed and these show that, compared to the parent (~4.5x the H_2_ produced after 3 h when 150 mM NaCl is added) and ΔMbhABC (~3x the H_2_ produced) strains, H_2_ production in the ΔMrp strain is less stimulated by the presence of Na^+^ (~2x the H_2_ produced; Supplementary Figure S5); thus, Mrp likely uses Na^+^ as a coupling ion for H^+^ pumping.

The results therefore show that the *P. furiosus* group 3 stand-alone Mrp is involved in the pH dependence of cell growth. H_2_ production assays using cell suspensions show that, like MBH, Mrp uses Na^+^ as the coupling ion. As the pH drops, Mrp brings in Na^+^ in exchange for pumping protons out of the cell in order to maintain cytoplasmic pH near optimum levels for growth. The marine environment inhabited by *P. furiosus* can be taken advantage of by Mrp, with close to 500 mM Na^+^ ions (compared to 10 μM protons at pH 5) available to balance pH by pumping protons out. However, this pH homeostasis by Mrp comes at a cost to ATP synthesis, as Mrp “competes” for Na^+^ influx with the ATP synthase of *P. furiosus*. This is best seen in the growth rates of the ΔMrp and parent strains in media with and without buffer. In the standard unbuffered medium, the parent strain has a slower growth rate than ΔMrp because it attempts to correct for the changes in pH that occur as growing cells produce CO_2_ during glycolysis. ΔMrp is not able to utilize the Na^+^ gradient to maintain pH, so all Na^+^ flux goes through ATP synthase and the cells quickly lyse upon reaching low pH. In buffered medium, the parent strain no longer requires Mrp to maintain pH, so the growth rate is faster than that of ΔMrp. This suggests that Mrp is able to act in the reverse direction, exchanging Na^+^ inside the cell for protons on the outside and contributing to the Na^+^ gradient utilized by ATP synthase, when pH is able to be maintained by a different mechanism, such as additional buffer in the medium. In fact, the results of Na^+^-dependent H_2_ production assays, which showed that the ΔMrp strain is more sensitive to changes in Na^+^ concentration than the parent suggests that, under optimal pH conditions, Mrp has a greater contribution to the Na^+^ gradient than the sodium pumping module of MBH.

## Conclusion

As a marine organism, *P. furiosus* is specifically adapted to life under high salt concentrations. However, while the stand-alone Mrp may contribute to some extent to the Na^+^ gradient, its primary function is not to maintain Na^+^ homeostasis inside the cell, as deletion of Mrp does not affect the range of Na^+^ concentrations that support growth. This is not the case with Mrp complexes involved in Na^+^ homoeostasis in other organisms. For example, expression of the group 1 Mrp from the halophile *Halomonas zhaodongensis* in *Escherichia coli* increases the range of suitable Na^+^ concentrations from 100 mM to almost 1 M NaCl (Meng et al., 2014). However, the genome of *P. furiosus* encodes a number of predicted Na^+^ pumping proteins (Supplementary Table S3), including four homologs of the single subunit NapA Na^+^/H^+^ antiporter, one copy of the single subunit NhaC Na^+^/H^+^ antiporter, a Ca^+^/Na^+^ cation antiporter, the sodium-dependent *acr*3 arsenical resistance gene, and multiple amino acid symporters and bacterial homologs of Na^+^-dependent neurotransmitters (Yamato and Anraku, 1990; Ito et al., 1997; Androutsellis-Theotokis et al., 2003; Furrer et al., 2007; Haja et al., 2020). *Pyrococcus furiosus* likely has a number of redundant systems in order to maintain cellular Na^+^ homeostasis in a high-salt environment but these do not include the stand-alone Mrp.

The results reported here with the ΔMbhABC strain provide additional insights into the function of MBH. While the growth rate of the ΔMbhABC strain was slower than that of the parent strain, the specific H_2_ production rates were the same for both strains, suggesting that hydrogenase activity in MBH is not directly coupled to Na^+^ pumping. This is consistent with the idea that the redox activity of MBH is directly coupled to the formation of a H^+^ gradient across the membrane and that this is passively exchanged by the Na^+^ pumping module in order to generate a gradient for ATP generation (Pisa et al., 2007; Yu et al., 2018). However, in contrast to MbhABC, deletion of MbhL, the catalytic subunit of MBH, abolished growth (in the absence of elemental sulfur; Schut et al., 2012). Together these data show that the primary role of MBH in *P. furiosus* is not as an energy conservation mechanism but simply as a method for disposing of reductant generated by glycolysis. The data also support the theory that MBH and related respiratory complexes evolved from the coupling of a simple cytoplasmic NiFe hydrogenase with a membrane-bound Mrp antiporter (Schut et al., 2016).

## Data Availability Statement

The original contributions presented in the study are included in the article/Supplementary Material, further inquiries can be directed to the corresponding author/s.

## Author Contributions

DH and MA designed the experiments, analyzed the results, and revised and edited the manuscript. DH performed the experiments and wrote the first draft of the manuscript. All authors contributed to the article and approved the submitted version.

## Conflict of Interest

The authors declare that the research was conducted in the absence of any commercial or financial relationships that could be construed as a potential conflict of interest.

## Publisher’s Note

All claims expressed in this article are solely those of the authors and do not necessarily represent those of their affiliated organizations, or those of the publisher, the editors and the reviewers. Any product that may be evaluated in this article, or claim that may be made by its manufacturer, is not guaranteed or endorsed by the publisher.
