# Supplementary material for: pH Homeostasis and Sodium Ion Pumping by Multiple Resistance and pH Antiporters in Pyrococcus furiosus
Source: Front Microbiol. 2021 Aug 16;12:712104. doi: 10.3389/fmicb.2021.712104 (PMC8415708; doi:10.3389/fmicb.2021.712104)
Supplement: Supplementary file 1 [file Data_Sheet_1.PDF]

Frontiers in Microbiology

pH Homeostasis and Sodium Ion Pumping by Mrp in *Pyrococcus furiosus*

Dominik K. Haja and Michael W.W. Adams*

Department of Biochemistry and Molecular Biology,

University of Georgia, Athens, GA 30602

Running Title: *P. furiosus* Mrp

Supplementary Information

Supplementary Tables S1-S3, Supplementary Figures S1-S5

Table S1. Primers used in this study.

Primer	Sequence
ΔMrp UFR F	agagtggccagctcattagagagtgttggaggattagcgacaattacaattttctttcta
ΔMrp UFR R	cctctagaatgttctccttcctcccccccattacaagggc
ΔMrp DFR F	actcagctcactccattttcaatccgggtatttggatacttctgg
ΔMrp DFR R	gaggcatcagtgaggaatactagcccaagtattattccaatcattcctcc

Table S2. UniprotKB IDs used for multiple sequence alignments.

**Gene**	**UniprotKB ID**
PF1147	Q8U1Q8
PF1148	Q8U1Q7
PF1149	Q8U1Q6
PF1150	Q8U1Q5
PF1151	Q8U1Q4
PF1152	Q8U1Q3
PF1153	Q8U1Q2
PF1441	Q8U0Z0
PF1442	Q8U0Y9
PF1443	Q8U0Y8
PF1444	Q8U0Y7
PF1445	Q8U0Y6
PF1446	Q8U0Y5
PF1447	Q8U0Y4
PF1448	Q8U0Y3
PF1449	Q8U0Y2
PF1450	Q8U0Y1
PF1451	Q8U0Y0
PF1452	Q8U0X9
PF1453	Q8U0X8
PF1423	Q8U107
PF1424	Q8U106
PF1425	Q8U105

PF1426	Q8U104
PF1427	Q8U103
PF1428	Q8U102
PF1429	Q8U101
PF1430	Q8U100
PF1431	Q8U0Z9
PF1432	Q8U0Z8
PF1433	Q8U0Z7
PF1434	Q8U0Z6
PF1435	Q8U0Z5
PF1436	Q8U0Z4
BSU31660	O05227
BSU31650	O05228
BSU31640	Q7WY60
BSU31630	O05229
BSU31620	O05260
BSU31610	O05259
BSU31600	Q9K2S2

Table S3. Predicted single-subunit Na^+ transporters in *P. furiosus*.

GM number	Annotation	Predicted Function
PF1603	hypothetical Na+ antiporter	NapA/CPA2/Kef-type K^+ ion importer
PF1164	probable Na+/H+ antiporter	
PF0268	Na+/H+ antiporter homolog	
PF0275	Na+/H+ antiporter	
PF2032	conserved hypothetical protein	NhaC-like Na^+ antiporter
PF0350	putative cation antiporter	Ca^+/Na^+ antiporter
PF0429	putative proline permease	Amino Acid symporter
PF0514	d-alanine glycine permease	
PF1469	glutamate/aspartate transport protein	
PF0552	arsenical-resistance protein *acr3*	Arsenical resistance
PF0371	putative transporter	Na^+-dependent neurotransmitter transporter
PF1254	probable sodium dependent transporter	
PF1726	conserved hypothetical protein	

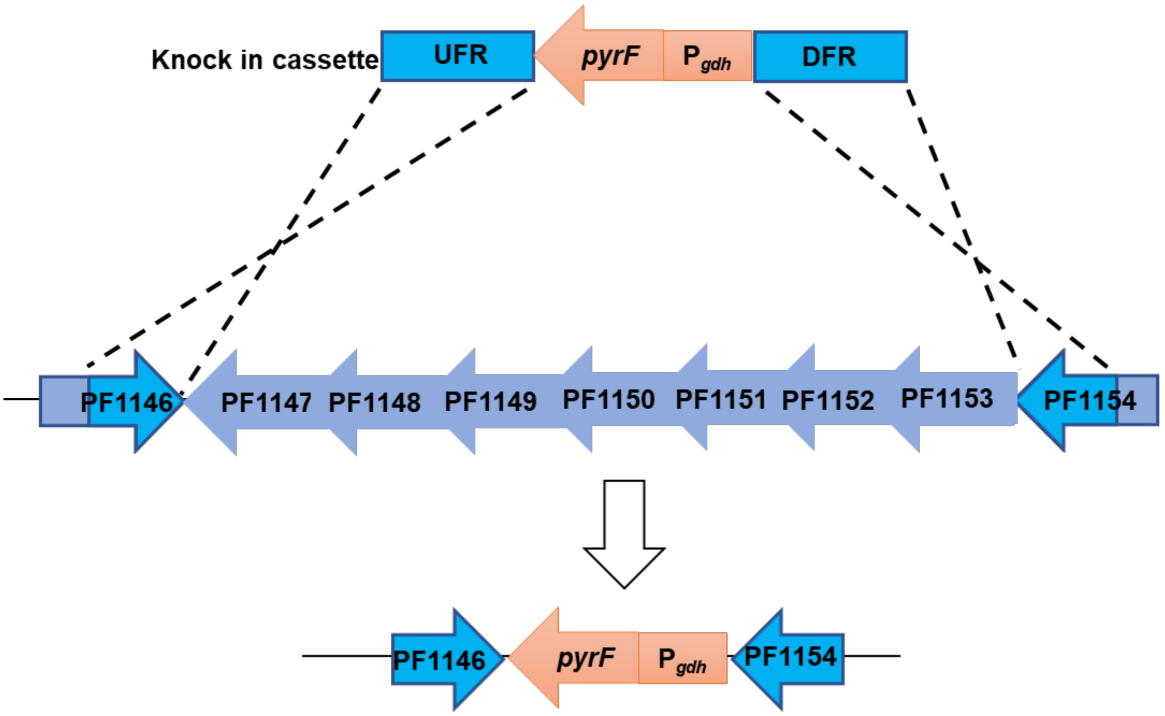

Figure S1: The genetic strategy used for construction of deletion strains. The knock-in cassette has homologous regions shown as the upstream flanking region (UFR) and downstream flanking region (DFR). The *pyrF* marker is under the control of the P_{gdh} promoter and allows for nutrient selection of transformants using uracil . The corresponding homologous loci of UFR and DFR are located in *Pf1146* and *Pf1154* for the deletion of Mrp. A similar strategy was used to delete *mbhABC*, using *Pf1422* and *Pf1426* as homologous loci for the UFR and DFR, respectively. Arrangement of the intergenic region after homologous recombination is also shown.

A

```
MbsH  MSQVAALLIALPLISAFFVPVLKQ-----IGKSLIKPFLVIITLLQTLIASWAFVQVYST 55
MbhH  -MTWLPFIIIIPLLGAFSMPIVSL-----LKGKAKEIWASMISFATLIVGIQVFREVWSK 54
MrpD  --MNASILALLP---AIFSLLIYLFGSLKIEGSIRARFKLPLTLEV--------KVLYLL 47
            ::  :*   *:   ::       :  .    :   :::            ::

MbsH  G--KPIIIYAGGWK-------PPIGINLYIGHFAALFILVIAVVSFLMALFNFKAVTV-E 105
MbhH  G--T--IVYTLGAPNPFGKATFPIRIVWEVDKFGALMVLIVTFVSFLAVIYSIEYMKHDT 110
MrpD  GVFLPVILLPFSQGGVVGGYSRLLGIEVDLDSITFLFLIAELTVFSMSSLYVVTYAT--- 104
      *     *:   .           : *   :. :  *:::    *  :  :: .   .

MbsH  PIDKYAMLFLLLLLGATGMIATGDIFNLFVFMEITAISAYALTAYNK-TGEAAEASMKYI 164
MbhH  GLEKFYTLILILELGMLGIAITGDIFNFYVFLEIMSIASYALVAFRNDTWEGIEAGIKYM 170
MrpD  EW-RKLSLLLLMHSGLIGAFISKDLFNFYVFMELSAISSFALIASSKEKGS-YKAAYNYL 162
         :   *:*::  *  *   : *:**::**:*: :*:::** *  : . .  :*. :*:

MbsH  VLGGIGSSFFLVGVALIYGATGTLNMAHLAMLANDINPTVVQVGLALIIFGLAVEAELFP 224
MbhH  FAGSLASSFVLLGIALLYGQYGTLTMGYLAVKIAENPTIVAKVALALFIGGLLFKSGAAP 230
MrpD  LLSMTASYLLVLSLGMIYFSTGTLNVKLAQG------REVPRIAVLIASLALSLKSGIFP 216
      . .  .* :.::.:.::*   ***.:             * ::.: :   .* .::   *

MbsH  LNAWAPDAYQAAPHPITVMFSAFVVKA-GLYAMARILYLF-KDVSGWSSLTKLLIAMATL 282
MbhH  VHMWLADAHPAAPSSISAMLSGLVIKIGGIYAIARIVFSIFSPTINLGTIGWIIIIFACI 290
MrpD  LHIWLPDAHSKADTYLSAILSGIAVKA-PVYGLVLLSYL--ADLS-------FLRPLAMM 266
      :: *  **:  *   ::.::*.:.:*   :*.:. : :              ::  :* :

MbsH  TVVFAELSALRQKNVKRMIAYSSIGQVGLIALALSLGTQ--------EGVSAGVFHMLNH 334
MbhH  TLIVGNAMAVVQEDLKRLLAYSSVGQIGYILLGLGIGMVAYGTRVGEIALAGAIYHTVNH 350
MrpD  SMIFGVVLAMVQVNAKRVLAYHTVSQMGYVLLAISYNNP----------YAAA-IYSFAH 315
      :::..   *: * : **::** ::.*:* : *.:. .             :..  : . *

MbsH  AIVKTMMFMAIGYVGITLGGTMIENFEGLGKRM----------PLTSLSLTIGGIATVGV 384
MbhH  ALMKALLFLVAGAVIHEIGTRNMNELSGLAKTM----------PKTTFAFLIGAAAIVGL 400
MrpD  AIFKSGLFLSIGALVDARKRKELNYLGCRNCYILLITVAILSLSIAGFGITVGGVAK--- 372
      *:.*: :*:  * :        :: :      :            : :.: :*. *

MbsH  PLFNVFWSKLRIILAAAHEGNLWPVALVLFASVVEAVYYFRLIHTMWFKGKSGERIPE-- 442
MbhH  PPLNGFASKWLIYESSALFNPILG-AIAVIGTVFCTAAYVRALFT--FFGRPSEKVTNAR 457
MrpD  ---------EILSKATKEEALIYGVSL-------GTAFSFAKLNYYLWKGYGHK------ 410
                 :  ::     :   ::        :.  .  :    : *   :

MbsH  ---GAIAIVLLLLAMLIIVIGVY--PTPFWNLV-TKA-------GSDIVEVSKYVANVLP 489
MbhH  DPGIAMMLPMIILVVTIIVMGFF--PWQISDRIMVPT-------ARALWDVIDYISSLMG 508
MrpD  -PSVKRVIPSLVLSLIALGMGIMWNGTPSYKDVLIPLGFVLFLLAKDYVPKRDYIIRVEP 469
             :  ::* :  : :*.        . :           .       .*:  :

MbsH  GVKL-------------- 493
MbhH  GG---------------- 510
MrpD  NYGVALLVLLISLFSIIT 487
```

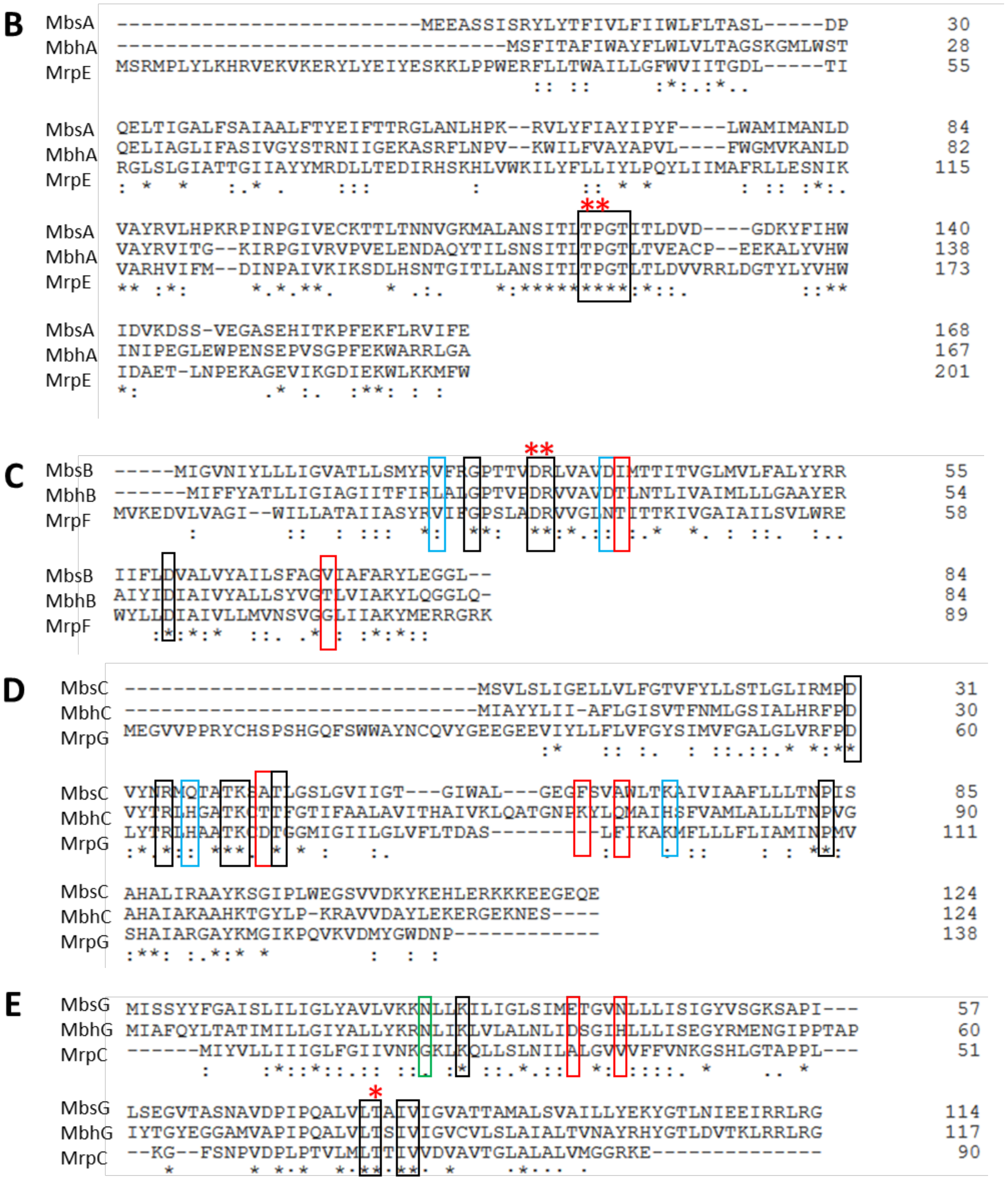
B
MbsA -------------------------MEEASSISRYLYTFIVLFIIWLFLTASL-----DP 30
MbhA --------------------------------MSFITAFIWAYFLWLVLTAGSKGMLWST 28
MrpE MSRMPLYLKHRVEKVKERYLYEIYESKKLPPWERFLLTWAILLGFWVIITGDL-----TI 55
MbsA QELTIGALFSAIAALFTYEIFTTRGLANLHPK--RVLYFIAYIPYF----LWAMIMANLD 84
MbhA QELIAGLIFASIVGYSTRNIIGEKASRFLNPV--KWILFVAYAPVL----FWGMVKANLD 82
MrpE RGLSLGIATTGIIAYYMRDLLTEDIRHSKHLVWKILYFLLIYLPQYLIIMAFRLLESNIK 115
MbsA VAYRVLHPKRPINPGIVECKTTLTNNVGKMALANSITITPGTITLDVD----GDKYFIHW 140
MbhA VAYRVITG--KIRPGIVRVPVELENDAQYTILSNSITITPGTLTVEACP--EEKALYVHW 138
MrpE VARHVIFM--DINPAIVKIKSDLHSNTGITLLANSITITPGTLTLDVVRRLDGTYLYVHW 173
MbsA IDVKDSS-VEGASEHITKPFEKFLRVIFE 168
MbhA INIPEGLEWPENSEPVSGPFEKWARRLGA 167
MrpE IDAET-LNPEKAGEVIKGDIEKWLKKMFW 201
C
MbsB -----MIGVNIYLLLIGVATLLSMYRVFRGPTTVDRLVAVDIMTTITVGLMVLFALYYRR 55
MbhB ------MIFFYATLLIGIAGIITFIRLALGPTVPDRVVAVDTLNTLIVAIMLLLGAAYER 54
MrpF MVKEDVLVAGI--WILLATAIIASYRVIRGPSLADRVVGINTITTKIVGAIAILSVLWRE 58
MbsB IIFLDVALVYAILSFAGVIAFARYLEGGL-- 84
MbhB AIYIDIAIVYALLSYVGTLVIAKYLQGGLQ- 84
MrpF WYLLDIAIVLLMVNSVGGLIIAKYMERRGRK 89
D
MbsC -----------------------------MSVLSLIGELLVLFGTVFYLLSTLGLIRMPD 31
MbhC -----------------------------MIAYYLII-AFLGISVTFNMLGSIALHRFPD 30
MrpG MEGVVPPRYCHSPSHGQFSWWAYNCQVYGEEGEEVIYLLFLVFGYSIMVFGALGLVRFPD 60
MbsC VYNRMQTATKSATLGSLGVIIGT---GIWAL---GEGFSVAWLTKAIVIAAFLLLTNPIS 85
MbhC VYTRLHGATKCTTFGTIFAALAVITHAIVKLQATGNPKYLQMAIHSFVAMLALLLTNPVG 90
MrpG LYTRLHAATKCDTGGMIGIILGLVFLTDAS--------LFIKAKMFLLLFLIAMINPMV 111
MbsC AHALIRAAYKSGIPLWEGSVVDKYKEHLERKKKEEGEQE 124
MbhC AHAIAKAAHKTGYLP-KRAVVDAYLEKERGEKNES---- 124
MrpG SHAIARGAYKMGIKPQVKVDMYGWDNP------------ 138
E
MbsG MISSYYFGAISLILIGLYAVLVKKNLLKILIGLSIMETGVNLLLISIGYVSGKSAPI--- 57
MbhG MIAFQYLTATIMILLGIYALLYKRNLIKLVLALNLIDSGIHLLLISEGYRMENGIPPTAP 60
MrpC ------MIYVLLIIIGLFGIIVNKGKLKQLLSLNILALGVVVFFVNKGSHLGTAPPL--- 51
MbsG LSEGVTASNAVDPIPQALVLTAIVIGVATTAMALSVAILLYEKYGTLNIEEIRRLRG 114
MbhG IYTGYEGGAMVAPIPQALVLTSIVIGVCVLSLAIALTVNAYRHYGTLDVTKLRRLRG 117
MrpC --KG--FSNPVDPLPTVLMLTTIVVDVAVTGLALALVMGGRKE-------------- 90

F

```
MbsD  -----------MNCIVCIEYIIVALMI-ISAILAVEWRDLLASTVGMAAVSLFASILFFF   48
MbhD  MHISKKKGVRKMNLDMIIQFIVLGGII-LSSVLMIVTRDLLVAVLASAAMSLLLSLEFYM   59
MrpB  -------------MDGTILEIITGIIAVTLALITVLHKKFLASLISYSLASLLLALLAMT   47
                       *   *: . :     ::: :  :.:*.: :. :   **: ::

MbsD  LQAPDVAMTEAAIGAALSAAVFIFAIKRTYRYETEEEEKLG-------------------   89
MbhD  LHAPDVAIAEAAVGAGVVTALVMYAISKTERWEREAP-----------------------   96
MrpB  FRAPDVALSLIVVGALV-IGLFIFAHEETREE-IKIDLKPGIAVIPLLLLLLKTRITPNS  105
      ::*****::   .:** :   .:.::* ..* .    :

MbsD  -----WWVRW----------------------------------------   94
MbhD  --------------------------------------------------   96
MrpB  LTYEAYLSVWNLGNLVTEILAGWRFYDSVGEALILFSAAVGFSIVVRRVK  155
```

G

```
MbsE  ------------------------------------------MVGMLKRVLA-ILTILVI   17
MbhE  ----------------------------------------------MKRALG-FLSLLVI   13
MrpB  MDGTILEIITGIIAVTLALITVLHKKFLASLISYSLASLLLALLAMTFRAPDVALSLIVV   60
                                                     *.    *:::*:

MbsE  G-------------------YWLAQGLADVPFGQ---DKMVV-GKYYLEHVKEETGAVNA   54
MbhE  FAS-----LL----VALSPEYGIKFGVGGEDWL----KYRYT-DNYYIEHGIEEVGGTNI   59
MrpB  GALVIGLFIFAHEETREEIKIDLKPGIAVIPLLLLLLKTRITPNSITYEAYLSVWNLGNL  120
                                  :  *:.           .  . ..  *    .  .  *

MbsE  VTAVVVNYRGLDTLGEVTVLFIASTGVAALLWKKKRERTAKTEGSVVLTTGARLLFPFIA  114
MbhE  VTDIVFDYRGYDTLGEATVLFTAIAGAVALLRPWRREENE--------------------   99
MrpB  VTEILAGWRFYDSVGEALILFSAAVGFSIVVRRVK-------------------------  155
      ** :: .:*  *::**. :** * .*   ::   :

MbsE  LFGMYIFIHGHLTPGGGFPGGATIATAFLLMYLAFTIYEIPHRGFEVTEGLAGMGYVITG  174
MbhE  ------------------------------------------------------------   99
MrpB  ------------------------------------------------------------  155

MbsE  LIGLAIGGYFLFDWIWQTWGWGHENIGRLFSGGFIPIIYTLIGIKVGTELSGIVDNMLKE  234
MbhE  ------------------------------------------------------------   99
MrpB  ------------------------------------------------------------  155

MbsE  EVKE  238
MbhE  ----   99
MrpB  ----  155
```

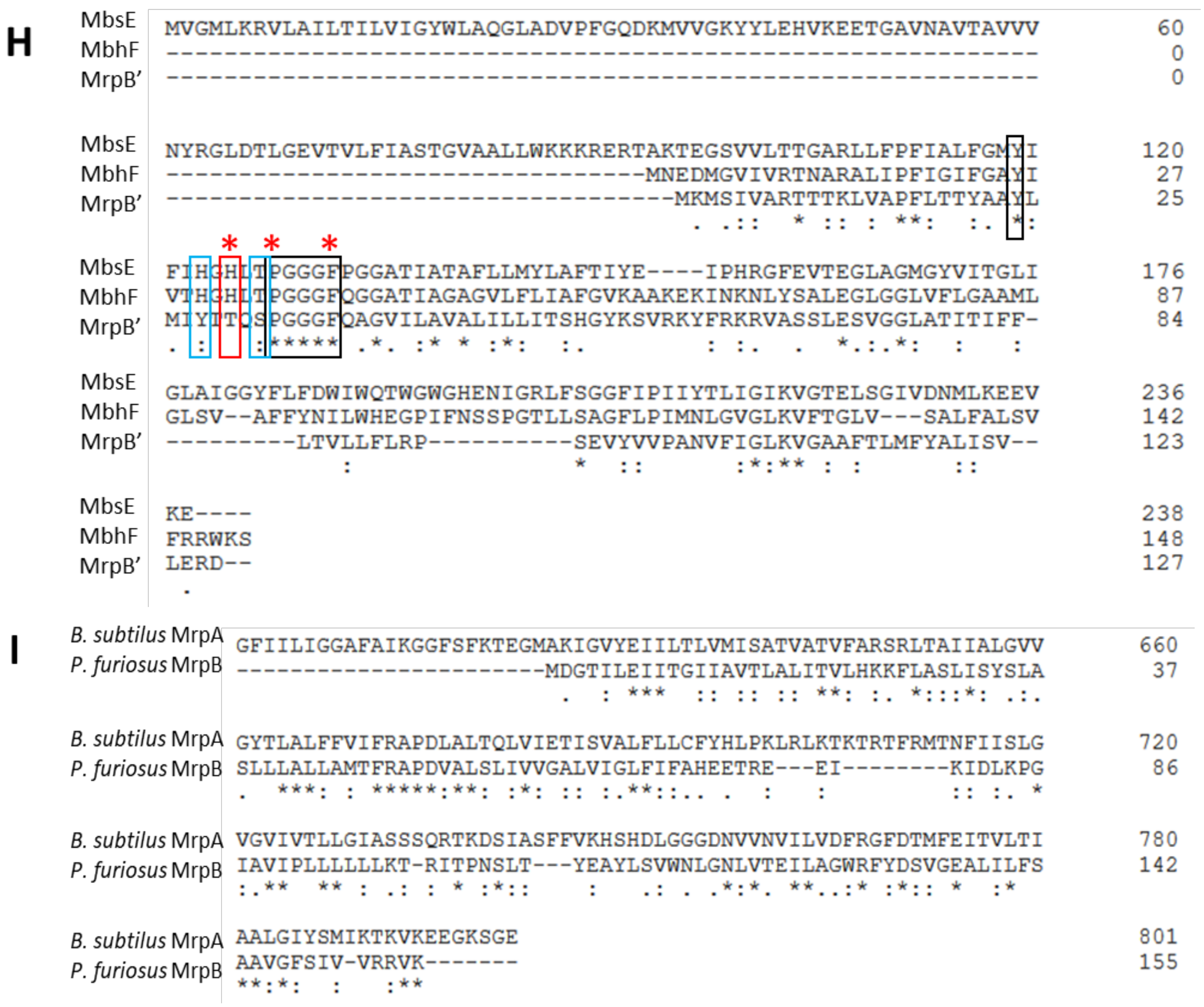

Figure S2. A-H: sequence alignments of *P. furiosus* Mrp subunits with homologous subunits in *P. furiosus* MBH and MBS; I: sequence alignment of *P. furiosus* MrpB and the C terminus of *B. subtilis* MrpA. Boxed residues represent proposed ion channels and conserved structural elements based on the MBH structure in (14). Black: identity; blue: high similarity; green: some similarity; red: not conserved. Similarity/identity is determined by properties of amino acid side chains, as calculated by Uniprot. Red asterisks represent amino acids with previously published mutations.

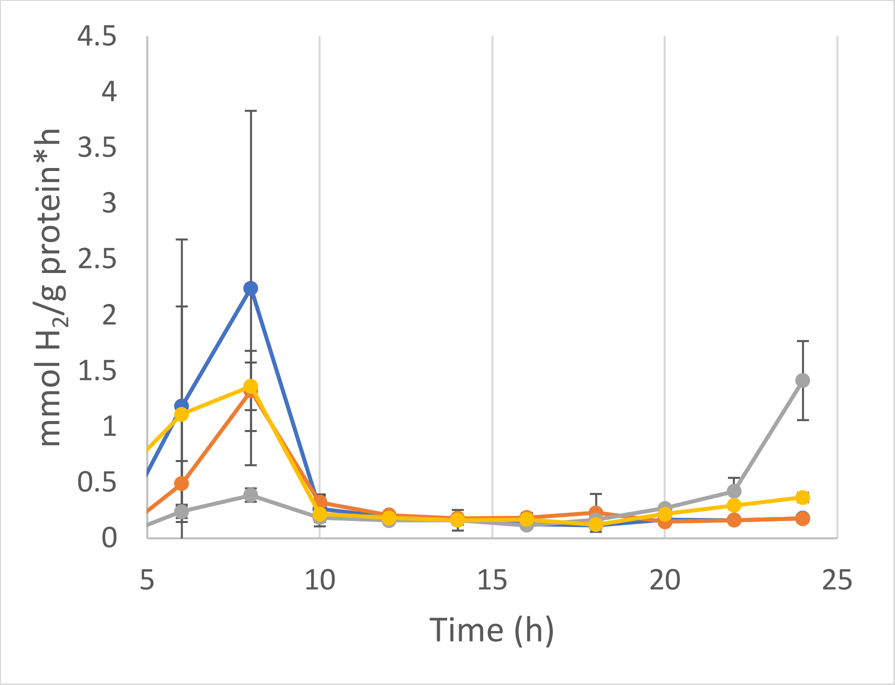

Figure S3. Calculated specific H_2 production rates corresponding to the growth data in Figure 1. Yellow, Parent strain; blue, ΔMbhABC; orange, ΔMrp; gray, ΔMrp/ΔMbhABC.

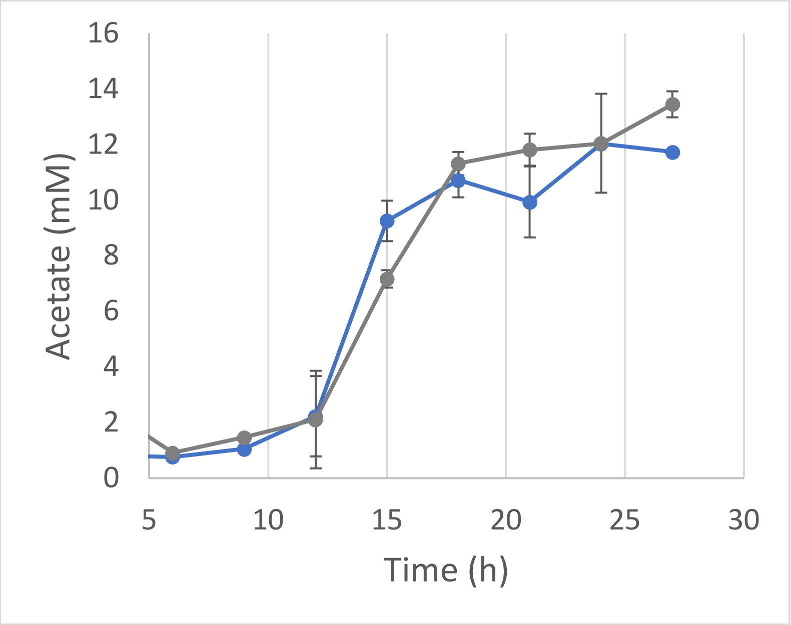

Figure S4. Acetate production of ΔMrp (gray) and the parent strain (blue) at pH 7. Corresponds to growth data in Figure 4. Error bars represent standard deviation from biological triplicate samples.

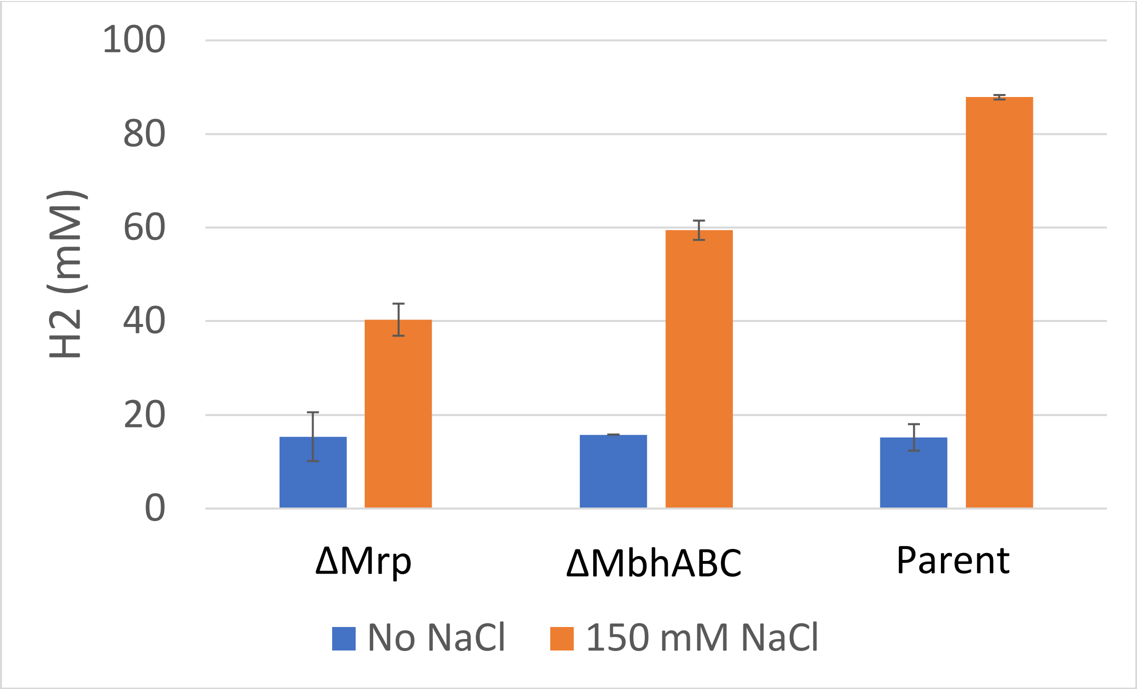

Figure S5. Na^+-dependent H_2 production in cell suspensions after 3 hours. Error bars represent standard deviation of technical triplicate samples.
